# Artificial intelligence-driven radiomics: developing valuable radiomics signatures with the use of artificial intelligence

**DOI:** 10.1093/bjrai/ubae011

**Published:** 2024-07-11

**Authors:** Konstantinos Vrettos, Matthaios Triantafyllou, Kostas Marias, Apostolos H Karantanas, Michail E Klontzas

**Affiliations:** Artificial Intelligence and Translational Imaging (ATI) Lab, Department of Radiology, School of Medicine, University of Crete, Voutes Campus, Heraklion, 71003, Greece; Artificial Intelligence and Translational Imaging (ATI) Lab, Department of Radiology, School of Medicine, University of Crete, Voutes Campus, Heraklion, 71003, Greece; Department of Medical Imaging, University Hospital of Heraklion, Heraklion, Crete, 71110, Greece; Computational BioMedicine Laboratory, Institute of Computer Science, Foundation for Research and Technology (FORTH), Heraklion, Crete, 70013, Greece; Department of Electrical and Computer Engineering, Hellenic Mediterranean University, Heraklion, Crete, 71410, Greece; Artificial Intelligence and Translational Imaging (ATI) Lab, Department of Radiology, School of Medicine, University of Crete, Voutes Campus, Heraklion, 71003, Greece; Department of Medical Imaging, University Hospital of Heraklion, Heraklion, Crete, 71110, Greece; Computational BioMedicine Laboratory, Institute of Computer Science, Foundation for Research and Technology (FORTH), Heraklion, Crete, 70013, Greece; Artificial Intelligence and Translational Imaging (ATI) Lab, Department of Radiology, School of Medicine, University of Crete, Voutes Campus, Heraklion, 71003, Greece; Department of Medical Imaging, University Hospital of Heraklion, Heraklion, Crete, 71110, Greece; Computational BioMedicine Laboratory, Institute of Computer Science, Foundation for Research and Technology (FORTH), Heraklion, Crete, 70013, Greece; Division of Radiology, Department for Clinical Science, Intervention and Technology (CLINTEC), Karolinska Institutet, Huddinge, 14152, Sweden

**Keywords:** radiomics, quantitative imaging biomarkers, artificial intelligence, machine learning, reproducibility, standardization, deep learning, deep radiomics, large language models, convolutional neural networks

## Abstract

The advent of radiomics has revolutionized medical image analysis, affording the extraction of high dimensional quantitative data for the detailed examination of normal and abnormal tissues. Artificial intelligence (AI) can be used for the enhancement of a series of steps in the radiomics pipeline, from image acquisition and preprocessing, to segmentation, feature extraction, feature selection, and model development. The aim of this review is to present the most used AI methods for radiomics analysis, explaining the advantages and limitations of the methods. Some of the most prominent AI architectures mentioned in this review include Boruta, random forests, gradient boosting, generative adversarial networks, convolutional neural networks, and transformers. Employing these models in the process of radiomics analysis can significantly enhance the quality and effectiveness of the analysis, while addressing several limitations that can reduce the quality of predictions. Addressing these limitations can enable high quality clinical decisions and wider clinical adoption. Importantly, this review will aim to highlight how AI can assist radiomics in overcoming major bottlenecks in clinical implementation, ultimately improving the translation potential of the method.

## Introduction

Radiomics, a rapidly growing field in medical imaging, offers a promising avenue for extracting quantitative features from radiographic images to inform clinical decision-making. Since radiomics endeavours to become integral to personalized medicine, the application of artificial intelligence (AI) holds immense potential in augmenting its capabilities. This article delves into the integration of AI techniques within radiomics workflows, elucidating their roles in enhancing data extraction, preprocessing, model development, and clinical implementation.

Historically, radiomics have faced challenges related to the standardization of imaging protocols, variability in image acquisition, the selection of segmentation methodology, the standardization of extracted features, the methods used for feature selection and the rigorous development and testing of predictive models using these features.^1^ This is the reason why quality scores such as the Radiomics Quality Score (RQS) was developed in 2017 to assist authors in avoiding methodological errors that can reduce the quality of radiomics analysis.^1^ Due to the lack of reproducibility of RQS,^2^ the European Society of Medical Imaging Informatics (EuSoMII) proposed in 2024 the METhodological RadiomICs Score (METRICS)^3^ as a tool that can assess the quality of radiomics studies and guide the development of high quality radiomics models. The integration of AI into radiomics holds the potential to address the issues and provide more robust and accurate analysis through advanced machine learning (ML) algorithms. Several steps within the process of radiomics analysis can benefit from AI integration, from image segmentation to feature selection^4^ and model development. Within the realm of data extraction and preprocessing, AI algorithms automate laborious tasks such as region of interest (ROI) segmentation and dimensionality reduction, while also facilitating the exploration of deep radiomics techniques. In model development, AI offers diverse methodologies tailored to the nuances of radiomics data, including traditional ML models, deep learning architectures, and Large Language Models (LLMs). Moreover, as radiomics advances towards clinical implementation, AI-driven approaches can automate processes address critical bottlenecks surrounding validation, reproducibility, and generalizability, thereby accelerating the integration of radiomics into routine clinical practice. Through a comprehensive exploration of AI integration in radiomics research, this article aims to present the transformative potential of AI in reshaping the landscape of radiomics in medical imaging and personalized healthcare and overcoming current barriers in the clinical translation of radiomics.

## Steps of radiomics analysis where AI can be applied

The pipeline of radiomics analysis consists of a series of steps that allow the extraction of quantitative data from medical images and the subsequent use of the data for the characterization of disease processes. Radiomics analysis has certain peculiarities which have to do with the type of data used (radiomics features) which are different to the data used for deep learning (whole images) or for applications in other domains of medicine which may utilize clinical data. These peculiarities pertain to the types of features extracted, the methodology for feature extraction, the preprocessing of the images to get quality features, the segmentation of regions of interest to extract features, the selection of relevant features for model building and the use of suitable algorithms for this type of data.^5^ These issues are unique to radiomics analysis and cannot be found in other types of AI applications encountered in other domains of medicine dealing with different types of data. Radiomics analysis starts from the construction of a suitable dataset of medical images and the preprocessing of these images to ensure image standardization and homogenization prior to radiomics data extraction. Once the dataset has been constructed and the images have been adequately prepared, a ROI needs to be segmented to mark the area where quantitative radiomics data will be extracted from. Radiomics features are then extracted from the selected ROIs and the dataset is subjected to a series of preparation steps to ensure that robust and informative features are used for further analysis. This data preparation step includes the exclusion of collinear features, the evaluation of feature robustness to segmentation and other sources of variability and the selection of the most informative features for model building. Finally, the dataset can be used for predictive model development. AI methods can be used in the majority of the aforementioned radiomics pipeline steps.

**Figure 1.**
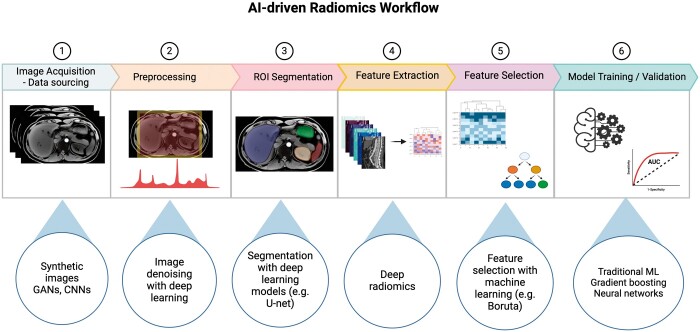
Steps of the radiomics pipeline where AI can be applied. From image acquisition to model training and validation all steps where AI can be used are explained and examples of potential uses are given in the bubbles below each step (created with biorender.com).

Standardizing the acquisition and reconstruction of medical images can be achieved with deep learning. Generative adversarial networks (GANs) can be used for image translation, resolution enhancement, and contrast synthesis while preserving texture for radiomics analysis.^6^ Such models can enhance dataset homogeneity by generating synthetic images that mimic variations observed in real-world medical imaging datasets. Deep learning can be used for the extraction of radiomics features from medical images (deep radiomics)^7^ and for semi-automated or automated ROI segmentation.^8^ ML models can also be used for efficient feature selection in an attempt to reduce the risk of overfitting^9^ and to ensure robust model generalization.^10^ Finally, AI can be used for the development of predictive classification^11^ or regression models using radiomics features. The following section with describe in detail how AI can enhance each of these steps of the radiomics pipeline. An overview of these steps and the contribution of AI can be seen on Figure 1.

## AI for data acquisition and preprocessing

### AI algorithms for data acquisition and preparation

In radiomics studies, the precision of initial steps is fundamental to the reliability and applicability of outcomes. The process begins with the acquisition of medical images using standardized protocols to maintain consistency across studies and ensure comparability.^12^ There’s a preference for single modality radiomics to prevent the complexities and potential overfitting associated with multi-modality data, unless multi-modality approaches demonstrate clear superiority.^3^ It is crucial that acquisition parameters, like slice thickness, contrast agent use, and imaging resolution, align with current clinical practices for the findings to be practically useful.

Biomedical images, obtained through diverse modalities such as MRI, CT, and PET scans, inherently exhibit variability due to differences in acquisition protocols and equipment characteristics and patient factors.^13^ Standardizing the acquisition and reconstruction processes is paramount to mitigate this variability. Here, AI interventions can prove instrumental: deep neural networks (DNNs) facilitate the generation of synthetic images, thereby ameliorating the effects of MRI acquisition variations. DNNs, particularly convolutional neural networks (CNNs), can learn complex patterns and variations within medical images. By training on diverse datasets, CNNs can adapt to variations in acquisition techniques, equipment, or patient-related, thus mitigating inherent variability in biomedical images. Architectures like GANs and UNet are adept at image translation,^14^ resolution enhancement, and contrast synthesis.^15^ It has been shown that denoising of CT images can increase the reproducibility of radiomics features.^16^ Additionally, these models enhance dataset homogeneity by generating synthetic images that mimic variations observed in real-world medical imaging datasets.

### AI algorithms for the automated ROI segmentation

Convolutional neural networks used for segmentation, such as U-Net, have emerged as powerful tools for semantic segmentation tasks in medical imaging.^17^ U-Net architectures demonstrate remarkable accuracy and reproducibility in ROI segmentation, comparable to or even surpassing manual delineation methods. Moreover, CNNs facilitate accurate contour propagation during image registration, enhancing longitudinal analysis by ensuring consistency across multiple time points. V-Net excels in semantic segmentation and detection tasks, particularly in volumetric image segmentation on CT and PET images. The V-Net architecture, a three-dimensional (3D) CNN, exhibits superior performance in capturing intricate anatomical structures and lesion boundaries,^18^ crucial for precise ROI delineation. Moreover, cascading 2 V-Nets to form a W-Net architecture enhances segmentation accuracy, particularly for bone-specific lesions, by leveraging the strengths of multiple networks.^19^

In essence, ML techniques, particularly segmentation CNNs like UNet and V-Net architectures, can revolutionize ROI segmentation in radiomics analysis. By automating segmentation processes, these algorithms streamline workflow, reduce interobserver variability, and enable robust feature selection for comprehensive characterization of imaging data.^20^ The integration of ML in ROI segmentation underscores its significance in advancing medical imaging and facilitating accurate diagnosis and treatment planning in clinical practice. Deep learning based organ segmentation with tools such as the TotalSegmentator^21^ and Multiple-Organ Objective Segmentation^22^ can be used for the construction of open access large scale radiomics datasets.^23^

### AI algorithms for dimensionality reduction and feature selection

Feature selection is the process to strategically balance the extracted features ability to tackle a specific task, while maintaining model simplicity and effectiveness.^24^ Effective feature selection is essential for avoiding overfitting, enhancing model interpretability, and ensuring the practicality of AI models in radiology. First of all, determining the appropriate number of features is a crucial, often overlooked initial step. This determination could be based on achieving similar performance across training and test datasets, employing statistical methods to identify the optimal feature count, consulting relevant literature. Alternatively, one heuristic rule suggests having 10 samples for each feature^25^ or *n*^2^ cases in the minority class for every *n* features considered, which can help in maintaining a balance between model complexity and predictive performance.

The application of unsupervised learning techniques, such as clustering or Principal Component Analysis (PCA), in feature selection is nuanced. While these methods can reveal data structure or reduce dimensionality,^26^ they do not directly align with the predictive or classification goals typical of supervised learning tasks. Their contributions, although valuable for insight, need careful consideration against the specific aims of a radiomics project.

Filter methods evaluate features based on their statistical characteristics, like their correlation with the target variable. The Pearson correlation coefficient is a widely used filter method that identifies features with a strong linear relationship to the target, discarding those with weak correlations. In contrast, the Minimum Redundancy Maximum Relevance method offers greater versatility. It is well-suited for multivariate analyses and accommodates various data types, unlike Pearson, which is tailored for bivariate analyses of continuous variables.^27^

Wrapper methods, such as Recursive Feature Elimination and Stepwise Selection, assess subsets of features based on a model’s performance. They provide insights into the interactions of features within the context of a predictive model. Although computationally demanding, they can identify the most effective feature combinations due to their model-centric approach. Innovative ML approaches like Boruta are also emerging.^28^ Boruta enhances the wrapper concept by incorporating the Random Forest (RF) algorithm with shadow features. This ML method evaluates the significance of actual features against random ones, offering a comprehensive and reliable feature selection mechanism.^29^

Embedded methods on the other hand integrate feature selection within the model training process, with Least Absolute Shrinkage and Selection Operator (LASSO) regression and Elastic Net being prime example. These methods penalize irrelevant features, thereby simplifying the model and reducing overfitting risk. LASSO, for instance, prunes the feature space by eliminating less important coefficients, retaining only impactful ones.^30^ Decision trees and their ensembles, like RF, inherently perform feature selection by selecting the most informative features for splitting data at each decision node, advantageous for managing complex datasets.

### AI algorithms to deal with class imbalance

Class imbalance is an important problem that can significantly affect AI algorithm results. This is even more important in small sample sizes where the low prevalence of one of the examined diseases can be more prominent, creating a bias of the predictions towards the more prevalent disease.^31^ To address this problem ML and more advanced AI algorithms can be employed. One of the most commonly used methods is Synthetic Minority Oversampling Technique (SMOTE).^32^ SMOTE creates synthetic samples of the minority class, increasing the number of instances of the minority class in the data, by creating new samples on the line between minority samples that are close to each other in the feature space.^32^ This strategy has been shown to alleviate bias related to class imbalance. Another approach that is commonly used is Adaptive Synthetic Sampling (ADASYN) which adaptively synthesizes minority samples by calculating a weighted distribution of various minority classes. ADASYN automatically decides how many synthetic cases to create in parts of the dataset where classification is difficult, compensating for scattered data, thus taking into account the peculiarities of the dataset. Ideally these techniques should not be applied to the whole dataset prior to the analysis but should be separately applied to the training set.^31^ Both SMOTE and ADASYN have been used in radiomics studies.^33–36^

Modern generative AI methods can be also used for data augmentation, alleviating problems related to class imbalance. GANs can be used for this purpose generating synthetic images to augment a dataset and have been shown to be used for state-of-the-art oversampling.^37^ Conditional GANs have been used to tackle class imbalance^38^ and has been also used in radiomics.^39^

### Deep radiomics as an alternative to handcrafted radiomics features

Deep radiomics integrate deep learning with radiomics to automate the extraction of feature representations directly from medical imaging data, thus eliminating the need for manual feature design. This approach relies on CNNs to analyse medical images and identify higher level abstractions that are predictive of various outcomes such as disease characteristics,^40^ treatment responses,^41^^,^^42^ and overall survival rates.^43^ The process begins with the use of a deep learning model, most commonly CNNs to extract features that are tailored to the specific dataset and clinical problem. ML techniques are then applied to select the most representative features, which are subsequently used in classification or regression models to address the question at hand.

CNNs are characterized by their ability to process data with grid patterns, such as medical images, and discern spatial hierarchies of features.^44^ Composed of convolution, pooling, and fully connected layers, CNNs are trained to minimize differences between outputs and ground truth labels through backpropagation and gradient descent. Unlike handcrafted radiomics, CNNs can be used to extract deep radiomics features eliminating the need for hand-crafted feature selection or expert segmentation.^45^ CNNs trained end-to-end have demonstrated superior performance over ImageNet pre-trained advanced networks in predicting radiomic features, particularly those related to lesion size and maximum/mean intensities, highlighting their potential for advancing diagnostic accuracy and clinical decision-making in radiomics.^46^ Deep radiomics spans several medical fields, including oncology (specifically GI, liver, and lung cancers), neurology,^47^ and cardiology,^48^ to support diagnosis, prognosis, and treatment planning. The goal is to advance personalized medicine by enabling more detailed and accurate patient assessments based on imaging data.

While deep radiomics share some limitations with handcrafted radiomics, such as the predominance of retrospective and uni-institutional studies and the potential lack of feature generalizability, it is distinguished by its robustness. Nonetheless, they require substantial data and computational resources, often necessitating GPUs for efficient processing. Additionally, deep radiomics lack in interpretability when compared to handcrafted radiomics. Such interpretability problems are extremely important for the clinical adoption of radiomics methods.^20^ To prevent overfitting, deep radiomics requires careful feature selection and can be further improved by incorporating clinical data, other omics data, or handcrafted radiomics, leading to models with enhanced performance.^49^

## AI for model development using radiomics data

### Traditional machine learning models utilizing tabular radiomics data

Traditional ML algorithms are commonly used in radiomics research, to build models using radiomics features. This section will focus on some of the most commonly used of these algorithms, presenting advantages and disadvantages of their use.

#### Support vector machines

Traditional ML models have emerged as powerful tools for analysing radiomics data in medical research and clinical practice. Among these models, Support Vector Machines (SVM) have demonstrated high stability and accuracy,^50^ outperforming linear regression, k-nearest neighbours, multilayer perceptron (MLP), and light gradient boosting machines in various metrics.^51^ SVMs adopt a nonparametric strategy, dynamically adjusting their parameters in response to the intricacies of the dataset they are trained on.^52^ SVM constructs decision boundaries (hyperplanes) between classes in an *n*-dimensional space (*n* represents the number of features for every sample in the database) and maximizes margins between these boundaries. In cases where the data is not linearly separable in the original feature space, SVMs use a kernel function to map the data into a higher-dimensional space where it becomes linearly separable. Common kernel functions include linear, polynomial, radial basis function, and sigmoid kernels.^53^ SVM classification models have been proposed in different clinical scenarios utilizing radiomics such as to distinguish between high-grade gliomas and low-grade gliomas, to distinguish between transient osteoporosis and avascular necrosis of the hip, to identify high risk endoleaks after aortic aneurysm repair, to detect lymph node metastases and many others.^54–56^

#### Random forests

Random forests are composed of multiple tree-structured classifiers, where each tree is built independently using a random vector k.^57^ The random vector k introduces variability into the tree construction process, ensuring that each tree in the forest is unique. During classification, each tree casts a unit vote for the most popular class at a given input. After generating a large number of trees, they collectively vote to determine the final class prediction. These models are effective in discovering interactions and non-linear effects of predictors, making them well-suited for analysing radiomics data.^58^ In fact, a study of tumour heterogeneity and angiogenesis properties on MRI, using a RF model has demonstrated high AUC and showed promise for non-invasive prediction of prognostic factors in breast cancer.^59^ However, RFs with deep trees and either no subsampling or excessive subsampling can be inconsistent.^60^

#### Gradient boosting

Gradient Boosting operates by sequentially adding weak learners, usually decision trees, to an ensemble, with each tree aiming to correct errors made by the previous ones.^61^ The model starts with a simple estimator representing the average of target values for regression or the most common class for classification. The weak learner’s predictions are combined with the ensemble’s predictions using a weight determined by a learning rate parameter. This process continues iteratively, with each new weak learner enhancing the overall predictive power of the ensemble by addressing the remaining errors. Ultimately, the final prediction is made by aggregating the predictions of all weak learners, weighted by their corresponding learning rates. This algorithmic framework has been applied to various radiomics scenarios such as the prediction of cardiovascular events and hospital readmissions.^62^ Regularization techniques such as subsampling, shrinkage, and early stopping mitigate overfitting. Tools like relative variable influence and partial dependence plots offer insights into variable importance and their effects on response, augmenting gradient boosting’s potential in extracting valuable insights from medical data.^63^ Gradient Boosting algorithms like Extreme Gradient Boosting (XGBoost), LightGBM, and CatBoost are widely used due to their effectiveness in capturing complex relationships in the data.

Extreme Gradient Boosting is an implementation of gradient boosting that is highly optimized and efficient. It constitutes an ensemble learning method for tabular datasets, combines decision trees sequentially using gradient boosting. It allows configuration of hyperparameters such as the number of estimators, maximum tree depth, and minimum child weight.^64^ XGBoost models that integrate radiomic features and clinical information have shown exceptional performance, surpassing other prediction models like SVM, Generalized Linear Model, and k-nearest neighbour (k-NN).^65^ XGBoost has been used in a wide variety of clinical scenarios such as the prediction of IDH1 mutation status in gliomas^66^ and in cases of multi-omics integration scenarios, such as in radio-transcriptomics^67^ and radio-metabolomics analyses.^36^

Light Gradient Booster, particularly LightGBM, stands out as a fast, high-performance gradient boosting framework. LightGBM optimizes efficiency by utilizing a decision tree algorithm based on histograms and Gradient-based One-side Sampling, which reduces time and memory usage compared to XGBoost. LightGBM’s leaf-wise algorithm with depth constraints enhances efficiency while preventing overfitting, contrasting with the level-wise decision tree growth strategies commonly used. Its ability to find meaningful node splits and impose a maximum depth limit leaf-wise further enhances efficiency and prevents overfitting.^68^ A study has shown that among 6 ML algorithms implemented (Linear Regression, RF, SVM, Linear discriminant analysis, LightGBM, and XGBoost.), LightGBM, alongside RF and XGBoost (XGB), emerged as optima ensemble learning techniques.^69^ The implementations of LightGBM in radiomics range from predicting papillary thyroid microcarcinoma,^70^ to estimating the haemorrhagic transformation risk in patients who suffered a stroke.^71^

CatBoost, proposed by Prokhorenkova et al., is an advanced refinement of the Gradient Boosted Decision Trees (GBDT) algorithm.^72^ Unlike traditional GBDT methods, CatBoost introduces innovations like the “Ordered Target Statistic” technique to efficiently handle high cardinality categorical variables.^73^ This method encodes categorical variables without risking target leakage, ensuring that no specific training example influences the encoding process. CatBoost excels in heterogeneous and categorical data settings although it may not be optimal for homogeneous or purely numerical datasets, as observed in biometric identification and activity recognition tasks. CatBoost is faster than alternatives like LightGradientBooster (LightGBM) and XGBoost and its automatic handling of categorical values and competitive performance make it a suitable choice for many applications, especially when considering the trade-off between speed and performance.^74^ Radiomics implementations of CatBoost include predicting fragility fractures^75^ and prostate cancer.^76^

In conclusion, traditional ML models, including SVM, RFs, and Gradient Boosting, hold significant potential for analysing radiomics data in medical research and clinical practice. These models offer high accuracy, stability, and flexibility in handling complex datasets, making them valuable tools for predicting prognostic factors and evaluating treatment outcomes. However, careful consideration of model parameters and interpretation of results are crucial for optimizing performance and ensuring clinical relevance.

### Deep learning models using radiomics data

Neural networks are increasingly being used in radiomics research. Compared to traditional ML algorithms, these approaches usually require more data to yield satisfactory results. The most important of these networks are presented in this section to demonstrate how deep learning can enhance radiomics analyses.

#### Artificial neural networks

Neural networks and deep learning have revolutionized medical image analysis, offering nuanced insights into complex patterns and features. Artificial neural networks (ANNs) have emerged as foundational tools in this domain by emulating the structure and function of biological neural networks, employing sigmoid functions and differentiable activation functions to facilitate learning by modifying weights. Neurons within ANNs receive multiple inputs, summing them and processing the sum through sigmoid functions to generate output values. Structurally, ANNs consist of layers—input, hidden, and output—transferring data via synapses, albeit the “black box” nature of hidden layers complicates interpretation. Learning in ANNs involves updating variables, adjusting connection strength between neurons, and enhancing output accuracy through backpropagation, wherein weights between neurons are adjusted based on errors between predicted and correct outputs. The gradient descent method minimizes errors by finding the lowest point in a cost function, streamlining the learning process of ANNs and enabling iterative adjustments for improved predictive performance across various tasks.^77^ Notably, use of the MLP ANN with back-propagation, has yielded promising outcomes, boasting high accuracy (90.97%), sensitivity (89.36%), and specificity (92.33%) in radiomics tasks. Moreover, an independent validation test demonstrated the model’s ability to generalize learning without overfitting, highlighting the potential of ANNs in unravelling intricate radiomic signatures.^78^ ANNs have been employed to predict cardiac event risk^79^ and to identify metastasis of tongue cancer.^80^

#### Recurrent neural networks

Recurrent Neural Networks (RNNs) are pivotal in radiomics for their capacity in processes like working memory and decision-making, which are crucial in analysing medical imaging data.^81^ RNNs are specifically designed for processing sequential or time-series data, featuring feedback connections that enable the retention of information from previous steps.^82^ Variants like Long Short-Term Memory (LSTM) and Gated Recurrent Unit (GRU) have evolved to address challenges like vanishing gradients, making them particularly suited for radiomics tasks where long-term dependencies in medical imaging data need to be captured effectively. Bidirectional RNNs (BRNNs) combine forward and backward RNNs, aiding in capturing context from both past and future sequences, which can be advantageous in analysing medical imaging sequences. RNNs find extensive application in radiomics, ranging from image classification^83^ and segmentation to disease diagnosis and prognosis prediction, leveraging their sequential processing capabilities to derive meaningful insights from medical imaging data. RNNs with radiomics, have been used to predict the progression of prostate cancer^84^ and classify breast cancer.^85^

#### Transformers

Transformers, shaped by self-attention mechanisms and pre-training on large corpora followed by fine-tuning, have not been widely used in radiomics research but offer significant potential in radiomics applications. Self-attention allows capturing long-range dependencies in sequences, crucial for tasks like language translation, while pre-training facilitates meaningful representation learning.^86^ Transformers, with their encoder-decoder structure and bidirectional representations, have significantly impacted language modelling and are starting to get implemented in medicine for detecting anomalies,^87^ segmentation and visualization tasks, particularly in brain tumour (BT) diagnosis and classification.^88^ They excel in capturing long-range spatial dependencies and utilizing whole-brain anatomical data, offering advantages over traditional CNNs. Visual transformers have been used to combine multimodal data, including radiomics and demographics, with MR imaging to predict the O6-Methylguanine-DNA methyltransferase status in patients with diffuse glioma.^89^ However, addressing challenges such as interpretability, limited datasets and high memory consumption remains essential for wider adoption in clinical settings and radiomics workflow.^90^

### Large language models in radiomics research

Large language models like GPT-3.5 and GPT-4 can handle complex textual data and perform natural language processing (NLP) tasks without extensive fine-tuning.^91^ These models are trained on vast amounts of text data, enabling them to recognize, interpret, and generate text with minimal or zero-shot learning properties. In healthcare, including radiomics, LLMs have shown promise in automating cognitive tasks traditionally performed by humans. Multimodal LLMs, such as Google’s Med-PaLM M and Microsoft’s LLaVA-Med, specifically designed for medicine, are being explored for clinical radiology applications,^92^^,^^93^ could potentially enhance radiomics analysis with their capabilities that integrate text, images, audio, and video data. Studies have demonstrated the efficacy of LLMs, including Bidirectional Encoder Representation from Transformers (BERT), BioBERT, and others, in extracting relevant information from radiology reports to predict clinical outcomes such as isocitrate dehydrogenase mutation in glioma patients, often outperforming or comparable to human readers.^94^ Examples of BERT models are: ViLBERT, LXMERT, and VisualBERT. Furthermore, the integration of NLP with radiomics facilitates the development of scalable pipelines for pain identification in cancer patients undergoing radiotherapy. By extracting pain scores from consultation notes and combining them with lesion-based radiomics features, these pipelines can enable efficient pain measurement, enhancing the overall diagnostic process in oncology.^95^ Nonetheless, drawbacks of LLMs such as the presence of hallucinations, necessitate caution when used for clinical applications including cases where radiomics are involved.^93^ Examples of studies with various model architectures can be found in Table 1.

**Table 1.** Examples of studies utilizing various types of AI architectures for data acquisition/preprocessing and predictive model development.

Topic	Author/year (^reference^)	Title	Aim of AI use	AI architecture(s)
AI for data acquisition and preprocessing	Ungan et al. (2022)^28^	Metastatic melanoma treated by immunotherapy: discovering prognostic markers from radiomics analysis of pretreatment CT with feature selection and classification	Feature selection and classification	SVM, Boruta, RF, LR, k-NN
	Zhao et al. (2021)^18^	Lung segmentation and automatic detection of COVID-19 using radiomic features from chest CT images	Segmentation	V-Net
	Xu et al. (2024)^14^	Synthesis of virtual monoenergetic images from kilovoltage peak images using wavelet loss enhanced CycleGAN for improving radiomics features reproducibility	Image generation	GAN
	Gitto et al. (2024)^35^	X-rays radiomics-based ML classification of atypical cartilaginous tumour and high-grade chondrosarcoma of long bones	Class imbalance correction	ADASYN
	Rich et al. (2021)^34^	Radiomics Predicts for Distant Metastasis in Locally Advanced Human Papillomavirus-Positive Oropharyngeal Squamous Cell Carcinoma	Class imbalance correction	synthetic minority over-sampling technique (SMOTE), ADASYN, and borderline SMOTE
	Ziegelmayer et al. (2020)^40^	Deep Convolutional Neural Network-Assisted Feature Extraction for Diagnostic Discrimination and Feature Visualization in Pancreatic Ductal Adenocarcinoma (PDAC) versus Autoimmune Pancreatitis (AIP)	Deep radiomics extraction	CNN, RF
AI for radiomics model development	Lee et al. (2022)^59^	Radiomic ML for predicting prognostic biomarkers and molecular subtypes of breast cancer using tumour heterogeneity and angiogenesis properties on MRI	Classification	naïve Bayes, linear regression, ANN, Decision Tree, RF
	Zhou et al. (2024)^50^	CT-Based Radiomics Analysis of Different ML Models for Discriminating the Risk Stratification of Pheochromocytoma and Paraganglioma: A Multicenter Study	Risk stratification (classification)	MLPs, SVM, RFs, k-NN
	Currie et al. (2019)^79^	Intelligent Imaging: Radiomics and ANN in Heart Failure	Risk stratification (classification)	ANN
	Klontzas et al. (2021)^56^	Radiomics and ML Can Differentiate Transient Osteoporosis from Avascular Necrosis of the Hip	Classification	XGboost, CatBoost and SVM
	Sakai et al. (2020)^66^	MRI Radiomic Features to Predict IDH1 Mutation Status in Gliomas: A Machine Learning Approach using Gradient Tree Boosting	Classification	XGBoost
	Chiari-Correia et al. (2023)^78^	A 3D Radiomics-Based ANN Model for Benign Versus Malignant Vertebral Compression Fracture Classification in MRI	Classification	MLP neural network with a back-propagation algorithm
	Sushentsev et al. (2023)^84^	Time series radiomics for the prediction of prostate cancer progression in patients on active surveillance	Classification using time series data	LSTM RNN
	Usuzaki et al. (2024)^89^	Identifying key factors for predicting O6-Methylguanine-DNA methyltransferase status in adult patients with diffuse glioma: a multimodal analysis of demographics, radiomics, and MRI by variable Vision Transformer	Classification using multimodal data	Vision transformer (vViT)

## How can AI overcome bottlenecks in the clinical implementation of radiomics?

### Establishing radiomics models of clinical value

A fundamental step towards the clinical acceptance of radiomics is establishing not merely its diagnostic performance but its tangible benefits on patient outcomes. This necessitates AI models that are designed not just for diagnostic accuracy but for practical clinical utility, such as enhancing decision-making processes in treatment planning or patient monitoring.^96^ Decision curve analysis, could be instrumental in demonstrating the clinical benefits of radiomics models.^97^ Importantly, the focus should be on conditions where the precision of the model can directly influence therapeutic decisions and outcomes. In this context, the role of radiologists becomes crucial; they not only interpret radiomic data but also integrate it with clinical insights to optimize patient care.

### Increasing the reproducibility of radiomics

Reproducibility and generalizability have been major stumbling blocks in radiomics^98^ and ML models in general.^99^ Studies often showcase models developed and tested in highly controlled environments, which may not perform equally well across different settings, scanners, or patient populations.^12^ By leveraging data from diverse sources, Radiomics models can be trained and tested for performance consistency, ensuring that they maintain accuracy and reliability regardless of variations in imaging protocols or equipment.

Radiomics faces a significant challenge with temporal variability, which can introduce variations in data even when the equipment, settings, and patient remain unchanged, exhibiting fluctuations in the same patient over time, highlighting the necessity for more rigorous test-retest studies to establish repeatability.^100^ While phantom studies offer a method to assess this variability, they may fall short in accurately emulating the complexity of human tissues, thus limiting their efficacy in fully capturing the nuances of temporal changes in radiomic features. This lack of repeatability undermines the reliability of radiomics, particularly in the context of delta-radiomics, where changes in radiomic features before and after treatment are analysed to predict outcomes such as response and survival.^101^

To tackle reproducibility issues in radiomics, implementing comprehensive solutions at different stages of image handling is essential.^102^ The adoption of AI-algorithms during image acquisition to standardize the imaging protocols will ensure consistency across different scanners and settings, crucial for minimizing initial data variability.^16^ Post-acquisition, image standardization is further refined using AI-driven techniques such as image denoising and brightness or contrast adjustment, which help to maintain the quality and comparability of the imaging data. Additionally, feature preprocessing methods like the ComBat algorithm^103^ or standardization algorithms play a pivotal role in correcting batch effects, applied directly on the radiomic data. Collectively, these approaches establish a comprehensive methodology for improving the consistency and utility of radiomics models in diverse clinical environments.

### Improving the translational potential of radiomics

AI offers targeted solutions to bridge the gap between controlled studies and real-world clinical applicability. First, to tackle the issue of data diversity and representation, AI can apply data augmentation and synthetic data generation, ensuring models are trained on datasets that more accurately mirror the patient population’s complexity,^104^ including those with comorbidities. Second, AI-driven bias mitigation algorithms can be employed to address the inherent selection bias and demographic imbalances, promoting a more balanced and equitable model performance. Last, the adoption of prospective, real-world datasets for model validation, facilitated by AI techniques such as transfer learning, ensures that models are not only theoretically sound but also practically viable in diverse clinical environments.^105^^,^^106^ By integrating these solutions, AI can effectively address the outlined problems, enhancing the reliability and real-world applicability of ML models in healthcare.

The comprehensibility of AI models is crucial for their endorsement by healthcare practitioners. This is particularly true for deep learning (DL) models, which are often perceived as more enigmatic and challenging to decipher by medical professionals. Radiologists and clinicians need to understand how models arrive at their conclusions to trust and effectively use them.^107^ Tools like Gradient-weighted Class Activation Mapping (Grad-CAM) and integrated gradients provide visual explanations of DL model decisions, highlighting regions of an image that influenced the model’s prediction.^108^^,^^109^ Shapley Additive exPlanations (SHAP) provide a sophisticated mechanism for highlighting the features within a radiomics dataset that are most critical for addressing a particular clinical issue. Additionally, SHAP quantifies feature influence on model outcome by assessing their weights within the algorithm.^110^ This transparency is essential for building trust and ensuring that AI-assisted decisions are aligned with clinical knowledge and experience.

Case-Based Reasoning could enhance radiomics interpretability by employing ΑΙ algorithms, for example, clustering to identify and retrieve similar previous patient cases. The idea focuses on demonstrating how the proposed model has analysed these cases and their outcomes, providing clinicians with a clear, relatable example of the AI decision-making process.^111^ This approach not only could elucidate the reasoning behind AI predictions but also anchor them in tangible clinical experiences.

**Figure 2.**
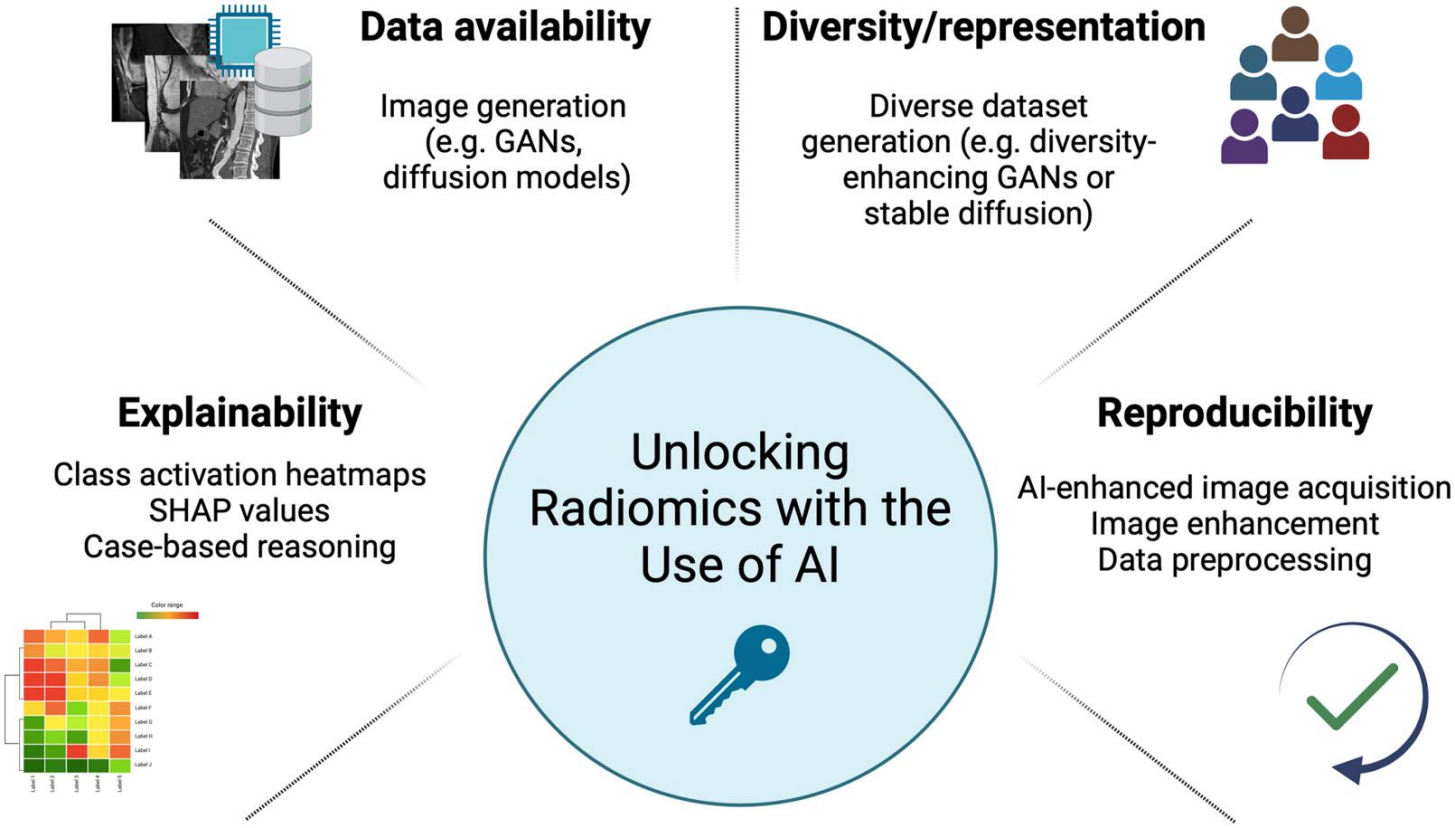
Bottlenecks of radiomics analysis which can be overcome by the use of AI. Data availability, diversity/representation, explainability, and reproducibility are the main bottlenecks that AI can alleviate (created with biorender.com).

Bottlenecks of radiomics analysis that can be overcome by radiomics are presented in Figure 2.

### Achieving high reporting standards for AI-driven radiomics

In the quest to increase the quality of published radiomics research, the use of reporting checklists is of utmost importance. Such checklists enforce and ensure the inclusion of all important information that will allow the reproduction of research results by researchers, while assisting reviewers of radiomics manuscripts.^112^ Radiomics-specific checklists have been developed including the CheckList for EvaluAtion of Radiomics research (CLEAR) which has been endorsed by the European Society of Radiology and EuSoMII.^113^ CLEAR covers all types of radiomics including AI assisted deep radiomics and ensures the sufficient reporting of all AI methods used in radiomics manuscripts. CLEAR can be used together with the METRICS score^3^ which has also been endorsed by EuSoMII for the evaluation of the quality of radiomics research. METRICS covers AI-assisted automated segmentation, deep radiomics and end-to-end deep learning solutions rendering it suitable for the assessment of manuscripts where AI methods have been used for the development of radiomics signatures. RQS had also been widely used in the past for the evaluation of radiomics research, but it has recently been shown that it lacks reproducibility.^2^

### Bias related to the use of AI in radiomics studies

Bias can be present at all steps of AI application, from study design to modelling and deployment.^114^ At the step of study design and data preprocessing, the use of AI in radiomics analysis can alleviate bias related to class imbalance, data preprocessing (eg, image normalisation) and feature selection, which can greatly affect the results of predictive models (discussed in detail in AI for model development using radiomics data section).^115^ Nonetheless, at the modelling step, the use of AI may be inherently linked to other types of bias which should be carefully avoided. These include data leakage and bias introduced by the selection of inappropriate evaluation metrics used to assess the performance of AI algorithms.^116^^,^^117^ To avoid such types of bias, feature selection, data normalisation and hyperparameter tuning should be performed at the training set and then the models should be tested at a completely unrelated hold-out internal test set and at an external test set.^117^^,^^118^ Finally, at the deployment step bias can be introduced by misuse of the algorithm for applications that it has not been trained on. Another important source of bias at this step is concept drift, where the relationship between the input and the output of the model changes for reasons such as changes in the reference standard (eg, the use of new guidelines) or changes in the equipment used to acquire images.^114^ Future research should focus on addressing all the aforementioned types of bias introduced by the use of AI in radiomics analysis.

## Conclusion

AI has the potential to significantly advance the clinical implementation of radiomics by addressing key challenges related to validation, reproducibility, and generalizability. By ensuring that radiomics models are validated against real-world data, designed with interpretability in mind, and integrated into clinical workflows, these models can move from being a promising research tool to becoming a cornerstone of personalized medicine. This transition will require close collaboration between AI researchers, radiologists, and clinicians to ensure that radiomic models are not only technically sound but also clinically relevant and trusted by those who use them to make critical healthcare decisions.
